# Acute Experimental Barrier Injury Triggers Ulcerative Colitis–Specific Innate Hyperresponsiveness and Ulcerative Colitis–Type Microbiome Changes in Humans

**DOI:** 10.1016/j.jcmgh.2021.06.002

**Published:** 2021-06-09

**Authors:** Jakob Benedict Seidelin, Martin Iain Bahl, Tine Rask Licht, Benjamin E. Mead, Jeffrey M. Karp, Jens Vilstrup Johansen, Lene Buhl Riis, Marina Ramírez Galera, Anders Woetmann, Jacob Tveiten Bjerrum

**Affiliations:** 1 Department of Gastroenterology, Herlev Hospital, University of Copenhagen, Copenhagen, Denmark; 2 Department of Clinical Medicine, University of Copenhagen, Copenhagen, Denmark; 3 National Food Institute, Technical University of Denmark, Kongens Lyngby, Denmark; 4 Harvard-MIT Division of Health Sciences and Technology, Institute for Medical and Engineering Science and Harvard Medical School, Cambridge, Massachusetts; 5 Koch Institute for Integrative Cancer Research, Massachusetts Institute of Technology, Cambridge, Massachusetts; 6 Harvard Stem Cell Institute, Harvard University, Cambridge, Massachusetts; 7 Broad Institute of MIT and Harvard, Cambridge, Massachusetts; 8 Institute for Medical Engineering and Science, Massachusetts Institute of Technology, Cambridge, Massachusetts; 9 Department of Chemistry, Massachusetts Institute of Technology, Cambridge, Massachusetts; 10 Ragon Institute of Massachusetts General Hospital, MIT, and Harvard, Cambridge, Massachusetts; 11 Harvard Medical School, Boston, Massachusetts; 12 Department of Anesthesiology, Perioperative and Pain Medicine, Brigham and Women’s Hospital, Cambridge, Massachusetts; 13 Bioinformatics Core Facility, Biotech Research and Innovation Centre, University of Copenhagen, Copenhagen, Denmark; 14 Department of Pathology, Herlev Hospital, University of Copenhagen, Copenhagen, Denmark; 15 Department of Immunology and Microbiology, University of Copenhagen, Copenhagen, Denmark; 16 LEO Foundation Skin Immunology Research Center, University of Copenhagen, Copenhagen, Denmark

**Keywords:** Acute Mucosal Injury, Innate Lymphoid Cells Type 3, ILC3, Innate Intestinal Response, Flare Initiation, Microbiome, Ulcerative Colitis, AMP, antimicrobial peptide, CD, Crohn’s disease, DAMP, damage-associated molecular pattern, GO, Gene Ontology, IBD, inflammatory bowel disease, IL, interleukin, ILC, innate lymphoid cell, MES, Mayo endoscopic subscore, NCR, natural cytotoxicity receptor, PAMP, pathogen-associated molecular pattern, qPCR, quantitative polymerase chain reaction, rRNA, ribosomal RNA, UC, ulcerative colitis

## Abstract

**Background and aims:**

The trigger hypothesis opens the possibility of anti-flare initiation therapies by stating that ulcerative colitis (UC) flares originate from inadequate responses to acute mucosal injuries. However, experimental evidence is restricted by a limited use of suitable human models. We thus aimed to investigate the acute mucosal barrier injury responses in humans with and without UC using an experimental injury model.

**Methods:**

A standardized mucosal break was inflicted in the sigmoid colon of 19 patients with UC in endoscopic and histological remission and 20 control subjects. Postinjury responses were assessed repeatedly by high-resolution imaging and sampling to perform Geboes scoring, RNA sequencing, and injury niche microbiota 16S ribosomal RNA gene sequencing.

**Results:**

UC patients had more severe endoscopic postinjury inflammation than did control subjects (*P <* .01), an elevated modified Geboes score (*P <* .05), a rapid induction of innate response gene sets (*P <* .05) and antimicrobial peptides (*P <* .01), and engagement of neutrophils (*P <* .01). Innate lymphoid cell type 3 (ILC3) markers were increased preinjury (*P <* .01), and ILC3 activating cytokines were highly induced postinjury, resulting in an increase in ILC3-type cytokine interleukin-17A. Across groups, the postinjury mucosal microbiome had higher bacterial load (*P <* .0001) and lower α-diversity (*P <* .05).

**Conclusions:**

UC patients in remission respond to mucosal breaks by an innate hyperresponse engaging resident regulatory ILC3s and a subsequent adaptive activation. The postinjury inflammatory bowel disease–like microbiota diversity decrease is irrespective of diagnosis, suggesting that the dysbiosis is secondary to host injury responses. We provide a model for the study of flare initiation in the search for antitrigger-directed therapies.

## Graphical abstract

**Figure:**
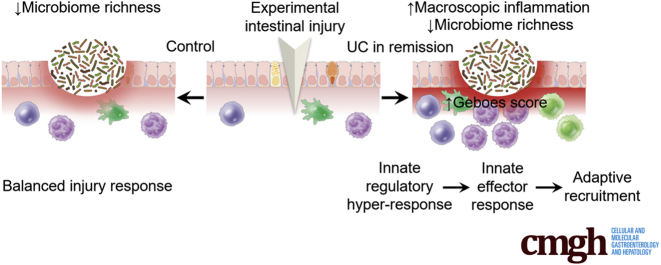

**Summary**

To explore mechanisms of flare initiation in ulcerative colitis, we used an in vivo intestinal mucosal injury model. Ulcerative colitis patients in remission have an increased postinjury macroscopic and histological intestinal inflammatory response caused by an underlying innate hyperresponse. Intestinal injury further decreases microbiome richness.

A prevailing hypothesis of ulcerative colitis (UC) flare initiation is that environmental factors —triggers—initiate sustained inflammation by causing an intestinal injury.^1^ This requires a preinjury high-risk state of dysfunctional barrier and immune function primed by genetic and epigenetic susceptibility enhancers. The preinjury high-risk state can be worsened further by risk modulating environmental changes such as dysbiosis resulting from early-life antibiotics exposure.^2^ In this increased risk state, triggers (eg, NSAIDs, emulsifiers, or gastrointestinal infections) are hypothesized to cause barrier breaches igniting a flare.^1^^,^^3^ The early responses to injury thus determine whether a trigger is harmful, as in UC, or can be contained, as in the healthy intestine.

However, our understanding of the normal and pathological response to intestinal mucosal injury in vivo in humans is less detailed than the knowledge on inflammatory aspects of active disease.^4^ This contrasts the detailed data on skin injury responses^5^ and might in part be due to limited use of suitable models for studying the mechanisms in humans. Investigating injury responses in vivo in humans would provide insight to the regulatory layers at play in early postinjury responses and the differences between these responses in the normal colon and the noninflamed UC colon. These differences could give a molecular explanation for the early phases of flare initiation and reveal potential targetable mechanisms to prevent disease exacerbations.

Multiple processes are engaged concomitantly in the acute phases after human skin breaks: epithelial cells dedifferentiate, migrate, and proliferate over the defect and respond together with stromal cells and residing immune cells to pathogen-associated molecular patterns (PAMPs) and damage-associated molecular patterns (DAMPs) liberated from injured cells and invading microorganisms. This activation both mounts innate responses to control the breach and recruits innate and adaptive immune cells to further augment this antimicrobial response.^6^ Innate lymphoid cells (ILCs) are pivotal for the initial orchestration of acute injury responses and important for later regeneration.^7^ Together with stromal signals, ILCs recruit neutrophils to the wound bed in response to PAMPs and DAMPs.^8^

ILCs are morphologically similar to T cells but lack antigen-specific receptors and can be divided into 3 groups, ILC1–3, based on their expression of transcription factors and cytokines.^9^ Data from mice show that while ILC1s are engaged in eliminating intracellular pathogens and viruses, ILC2s are important for helminth infection defense. ILC3s are, however, the most prevalent ILCs in the murine gastrointestinal tract, where they play an important role in innate responses towards invading pathogens through secretion of interleukin (IL)-17A and IL-22.^10^ ILC3-derived cytokines not only stimulate epithelial cells to produce antimicrobial peptides (AMPs) and chemokines attracting neutrophils,^11^ but also regulate adaptive and regulatory T cell responses of key importance for intestinal homeostasis in mice.^12^^,^^13^

A recent single-cell analysis of immune cell signatures in inflammatory bowel disease (IBD) showed that ILCs are present but not increased in the preinflamed mucosa of UC patients.^14^ Animal colitis models suggest that exaggerated ILC3 activation worsen experimental colitis through the secretion of IL-17A and IL-22 and subsequent excess neutrophil influx resulting in tissue damage.^15^ However, lack of IL-22 and impaired ILC3 function has been shown to aggravate experimental colitis and various microbial infections due to their role in barrier function maintenance and intestinal homeostasis (reviewed in Zhou and Sonnenberg).^16^

The invading microbes are killed by neutrophil phagocytosis^17^ and AMPs released from the neutrophils^18^ and activated epithelial and stromal cells, which are important contributors of AMPs like β-defensins, lectins, and cathelicidine in mice.^19^ This combined with decreases in tissue oxygenation due to the oxidative burst of neutrophils could change the microbiota.^18^ The microbiota has indeed been shown to be less diverse in both UC and Crohn’s disease (CD), with disease- and site-specific changes in compositions, and metabolomic profiles, which in theory could result in or from barrier defects and increased engagement of the immune system and inflammation.^20^ However, the IBD-related microbiota have also been regarded as a dysbiosis causing inflammation.^21^ The interplay between the microbiota and host in human intestinal injury is less investigated, and the causative relationship between microbiota changes and UC inflammation remains to be determined.^22^

Most of the observations regarding the nature and timing of responses to intestinal injury are thus based on in vitro and animal studies. Because essential differences exist between, for instance, murine and human immune responses, between microbial compositions, and to an even higher degree, between experimental animal colitis models and IBD,^23^ it is uncertain how murine findings applies to human injury responses. We therefore wanted to study the human acute response to a superficial mucosal injury of the colon.

The main aim was to characterize the early responses to an intestinal superficial barrier injury to determine if the physical injury trigger response differs in the normal and the uninflamed UC intestine. We therefore used a human in vivo intestinal injury model developed from the method devised by Anthony Segal’s group^24^ and earlier used by ourselves^25^ allowing repeated macroscopic imaging and injury site sampling over time. The aim was to follow the macroscopic and histopathologic injury responses over time and to identify transcriptomic postinjury host responses and concomitant changes in the injury site microbiota niche. Ultimately, our aim was to identify abnormalities in response to injury in UC that could be engaged in flare initiation.

## Results

### Macroscopic and Histological Wound Characteristics

**Figure 1:**
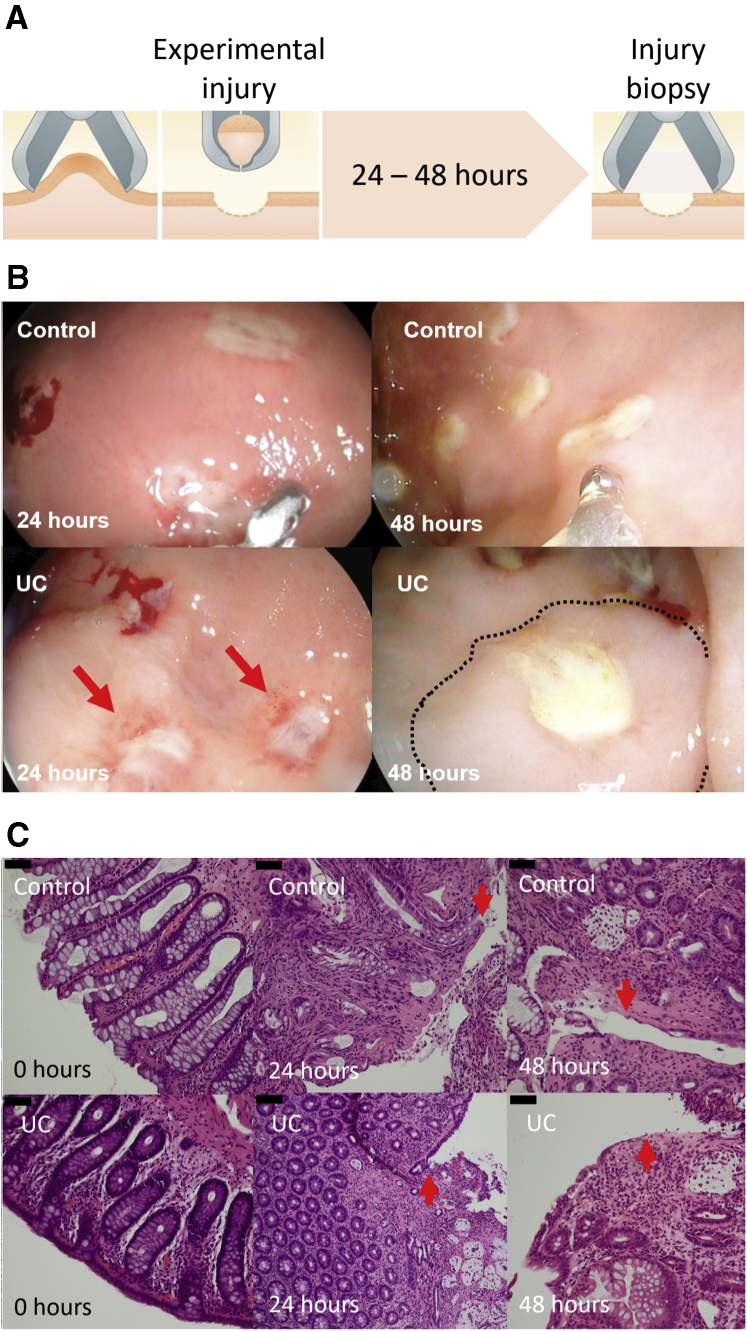
**Characterization of the human intestinal acute injury model.** (*A*) Schematic outline of the model. Wound biopsies were taken 24 and 48 hours after an initial injury. (*B*) Macroscopic appearance of the injured mucosa. An increased inflammation was seen with erythema and increased edema in UC. (*C*) Histologic appearance of the injury. An acute inflammation was seen in both control subjects and UC patients. The edges of the mucosal breaks are marked by red arrows.

**Figure 2:**
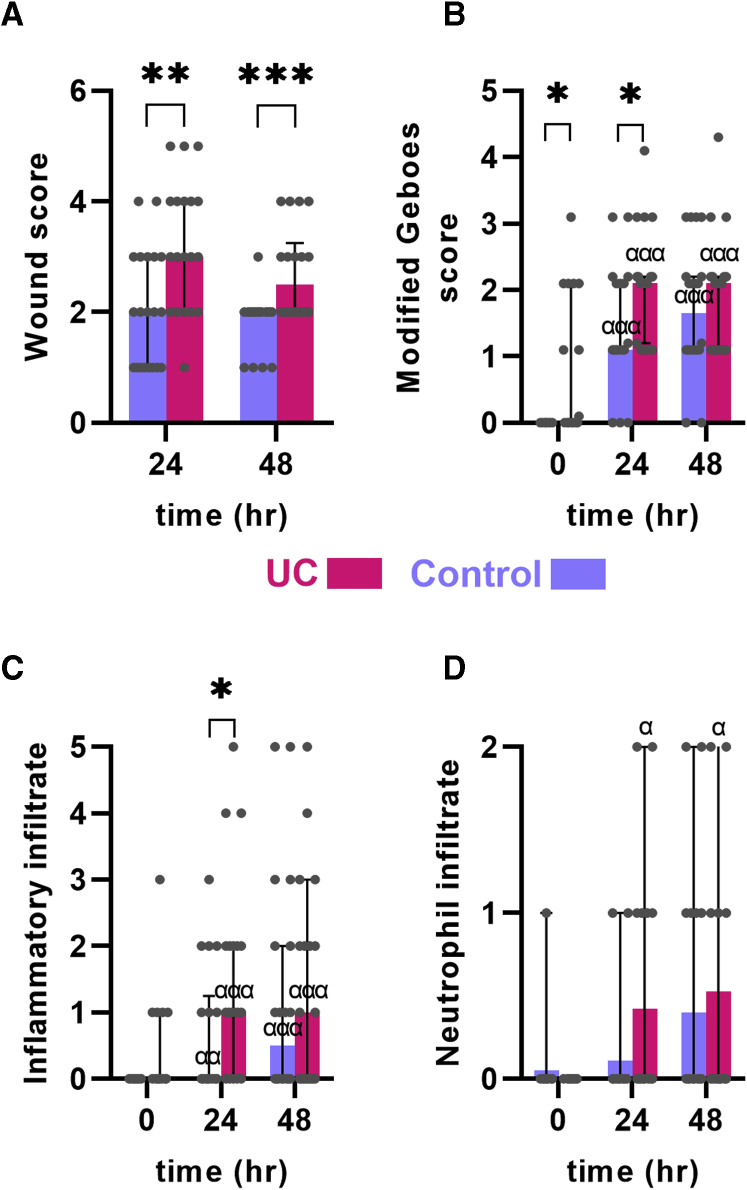
**Postinjury macroscopic and microscopic inflammation.** (*A*) A wound score of the macroscopic changes after mucosal injury (see text and Table 2 for elements of the score). UC patients had increased wound score compared with control subjects. (*B*) Inflammation assessed by the modified Geboes score (see text for explanation). UC patients had a more severe modified Geboes score. (*C*) Geboes inflammatory infiltrate subscore. (*D*) Geboes neutrophil infiltrate subscore. Medians and interquartile ranges are shown as well as individual values. Red: UC patients. Blue: control subjects. ∗*P <* .05; ∗∗*P <* .01; ∗∗∗*P <* .001; α*P <* .05 compared with preinjury of same diagnosis.

All participants were without endoscopic inflammation (ie, Mayo endoscopic subscore [MES] 0) (Table 1) at the initial endoscopy, and none developed general inflammation outside the wound area during the observation time. No complete healing of the experimental wounds was seen within the observation time. Generally, the wounds had 3 types of macroscopic coverage of the wound bed: no coverage and blood clotting above the wound, mucus or pus secretion covering the wound, or partial or complete coverage with a whitish thin layer of the wound bed. Further, 2 signs of inflammation were seen: disappearance of visible vessels in the mucosa adjacent to the wound (as a sign of edema) and hyperemia (Figure 1*B*). The wounds were scored based on these characteristics (Table 2, Figure 1*B*). Patients with UC, albeit in remission, had signs of more aggressive inflammation and less regeneration at both time points 24 and 48 hours after the injury based on the wound score (*P <* .01 and *P <* .001, respectively) (Figure 2*A*).

**Table 1:** Patient Characteristics

	UC Patients	Control Subjects
Patients	19	20
Female	12 (63)	12 (60)
Age, y	26 (21-63)	53 (33-70)
Debut <24 y	1 (5)	N/A
Disease duration >10 y	11 (58)	N/A
Tobacco use	0 (0)	2 (10)
Mayo endoscopic score (range)	0 (0-0)	0 (0-0)
Left-sided colitis	17 (89)	N/A
Pancolitis	2 (11)	N/A
Local mesalasine	0 (0)	N/A
Oral mesalasine	15 (79)	N/A
Azathioprine	2 (11)	N/A

Values are n, n (%), or median (range).

N/A, non-applicable; UC, ulcerative colitis.

**Table 2:** Colonic Mucosal Wound Healing Score

Wound	Score
Complete fibrin-like coverage	1
Partial fibrin-like coverage	2
No fibrin-like coverage (clotting only)	3
Inflammation	
Peripheral hyperemia	1
Edema	1
Total score	1-5

Histologically, the inflammatory pattern was an acute-type reaction with infiltration of neutrophils and eosinophils attracted to the wound area (Figure 1*C*). The experimental wounds were found to extend to—but not involving—the muscularis mucosa layer. When comparing the inflammatory pattern in UC patients and control subjects, a similar pattern to the macroscopic wound score was seen in the modified histological Geboes score: at 24 hours, this score was more elevated in UC patients than in control subjects (*P <* .05), whereas the modified Geboes scores were similar after 48 hours (Figure 2*B*). This difference was mainly driven by a higher inflammatory infiltrate subscore (*P <* .05 at 24 hours) (Figure 2*C* and *D*). As expected, patients with UC had a higher baseline modified Geboes score due to degenerative changes (crypt disruption) as a sign of previous inflammation.

### Regulatory Host Changes After Intestinal Injury

**Figure 3:**
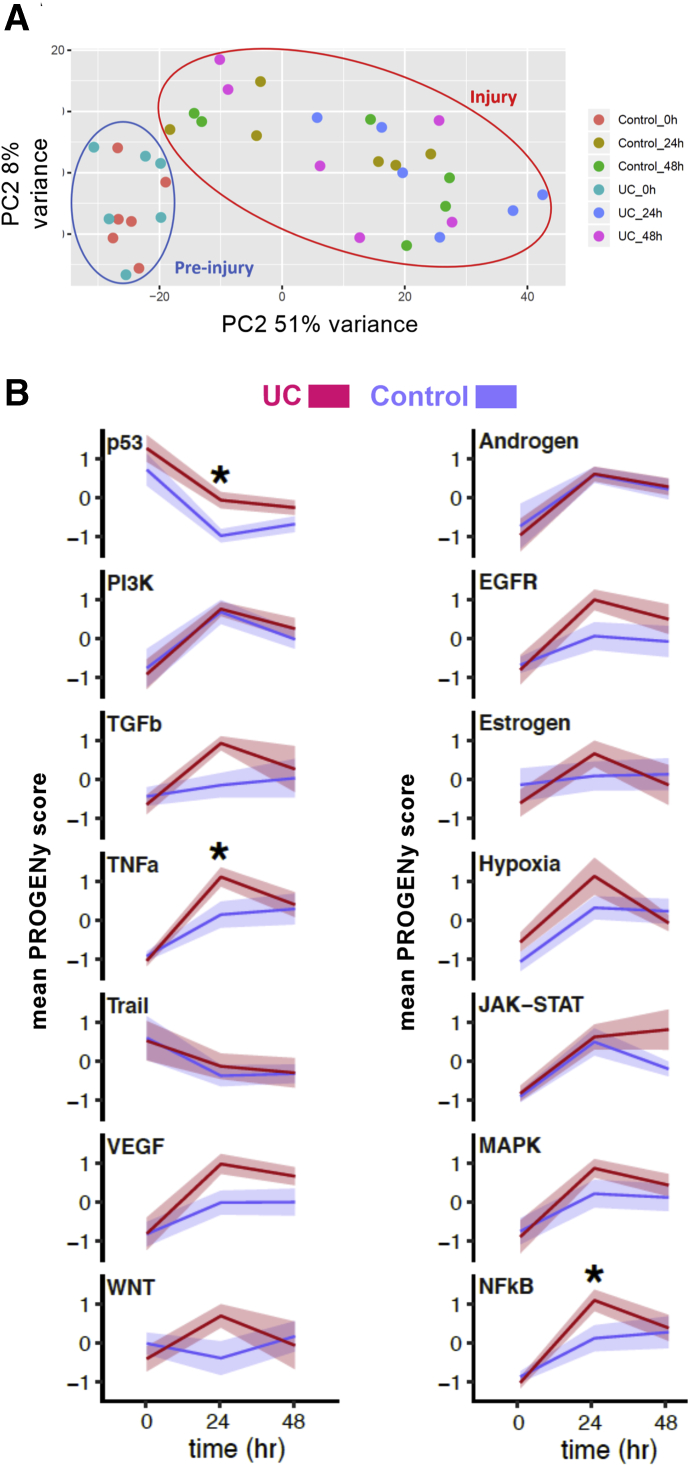
**Bioinformatic analyses of the RNA-sequencing data from host tissue.** (*A*) Principal component (PC) analysis of pre- and postinjury responses. Postinjury samples are grouped together. (*B*) PROGENy pathway inference analysis. The analysis identified the kinetics of the main signaling engaged in acute injury responses in the human colon on models including diagnosis (UC vs control) and time. Signaling pathway activity was inferred over 14 pathways using a diagnosis × time point model of the raw count data, mean pathway activity, and associated SEM is presented. Pairwise *t* testing with Benjamini-Hochberg multiple comparison correction was performed over all groups, with significant (∗adjusted *P* < .05) differences at each time point between UC patients and control subjects denoted.

To investigate the host responses to intestinal injury, RNA sequencing was performed on index control biopsies and subsequent wound biopsies. The initial principal component analysis revealed clustering of index biopsies irrespective of diagnosis and a separate clustering of the wound biopsies irrespective of diagnoses (Figure 3*A*).

To further explore dynamic differences to acute injury at the level of cell signaling pathways between UC patient and control subjects, PROGENy pathway inference was performed. This analysis identifies the kinetics of the main signaling engaged in acute injury responses in the human colon on models including diagnosis (UC vs control) and time. Fourteen regulated pathways were identified using this diagnosis × time point model (Figure 3*B*). A distinct pattern of early engagement nuclear factor kappa B, mitogen-activated protein kinase, and tumor necrosis factor alpha signaling in UC was seen (adjusted *P <* .05). Identification of the top enriched or depleted gene set enrichment analysis modules from the Broad’s MSigDB Hallmark collection as well as identification of top enriched Gene Ontology (GO) terms likewise revealed early engagement of inflammation and innate pathways in UC with early enhanced engagement of tumor necrosis factor alpha or nuclear factor kappa B, inflammatory response genes and IL-2 or signal transducer and activator of transcription 5, and IL-6 janus kinase or signal transducer and activator of transcription 3 signaling hallmark genes.

### Engagement of Postinjury Innate Responses

**Figure 4:**
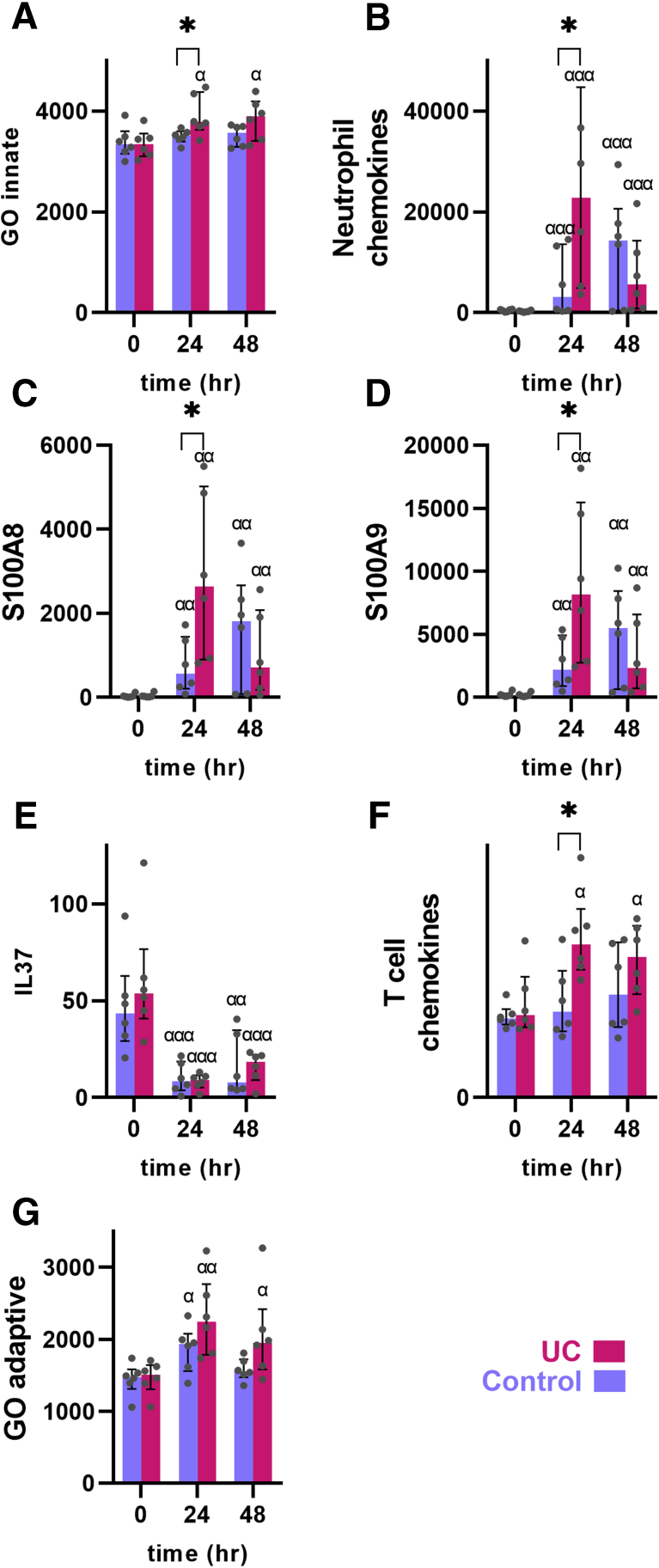
**Innate and adaptive immune responses in the acute injury model.** (*A*) Innate GO term gene set expression. UC patients had a more pronounced induction of innate related genes. (*A*) Expression of neutrophil chemokine genes showed rapid and increased expression in UC. (*C*, *D*) Expression of neutrophil-derived calprotectin subunits S100A8 and S100A9. These were induced more rapidly in UC postinjury. (*E*) Expression of the innate immune suppressor IL-37 was lowered in both UC patients and control subjects postinjury. (*F*) Lymphocyte-attracting chemokine gene expression was increased in UC. (*G*) Adaptive innate immune response GO term gene set expression. Medians and interquartile ranges are shown as well as individual values. Red: UC patients. Blue: control subjects. ∗*P <* .05; α *P <* .05, αα *P <* .01, ααα *P <* .001 compared with preinjury of same diagnosis.

Because macroscopic and histological assessment revealed an early and more exaggerated inflammatory postinjury response in UC, engagement of innate signaling was investigated. Indeed, a significant increase in innate response genes (GO:0045087) was found in UC patients compared with control subjects (*P <* .05 at 24 hours) (Figure 4*A*) and compared with preinjury levels (*P <* .05 for both 24 and 48 hours in UC). In line with the histopathological findings, neutrophil recruiting chemokines were intensely induced in UC patients (89.2-fold compared with preinjury) and much more than in control subjects (6.3-fold compared with preinjury; *P <* .001 at all time points postinjury; *P <* .05 comparing control subjects and UC patients at 24 hours) (Figure 4*B*). Looking at neutrophil markers S100A8/S100A9 confirmed the pattern being highly increased at 24 hours in UC patients (114.4-fold and 65.7-fold for 24 and 48 hours, respectively) compared with control subjects (31.9-fold and 14.3-fold, respectively; *P <* .05 at 24 hours; *P <* .01 at all time points postinjury) (Figure 4*C* and *D*). However, the kinetics of neutrophil engagement differed between UC patients and control subjects: in UC neutrophil markers were rapidly increased but decreased already after 48 hours, whereas the increase in control subjects was more modest and continued after 48 hours (Figure 4*B–D*). Interestingly, the innate suppressor cytokine IL-37 was greatly suppressed in both conditions postinjury, thus loosening the restrain on the innate immune system in response to injury (*P* < .01 at all time points postinjury) (Figure 3*E*). Apart from signs of early engagement of innate responses in UC, lymphocyte recruiting chemokines and adaptive immune responses were also seen (Figure 4*F* and *G*).

### Innate Response Regulating Cells

**Figure 5:**
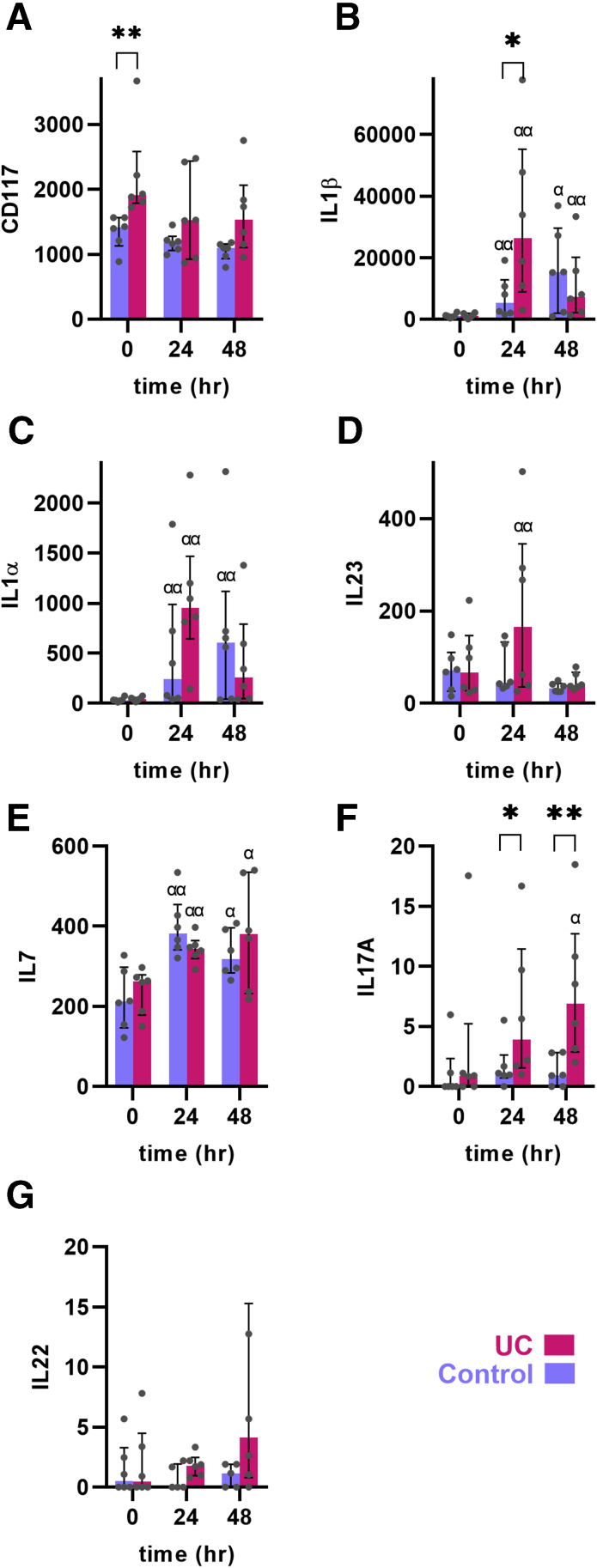
**Engagement of ILC3-type markers in the acute injury model.** (*A*) Expression of the ILC3 marker CD117 was increased preinjury in UC. (*B–E*) Expression of ILC3 activating cytokines IL-1β, IL-1α, IL-23 and IL-7. IL-1β was induced the most postinjury. (*F*, *G*) Expression of ILC3-type cytokines IL-17A and IL-22. IL-17A was significantly induced in UC. Medians and interquartile ranges are shown as well as individual values. Red: UC. Blue: control. ∗*P <* .05; ∗∗*P <* .01; α*P <* .05, αα*P <* .01 compared with preinjury of same diagnosis.

In order to explain the innate hyperresponse in UC an analysis of innate response regulating cells was performed. ILCs have in general been shown to be important for innate responses, but ILC3s more so as key directors of intestinal innate responses in mice. Indeed, the ILC3 marker CD117 was enriched preinjury in UC patients, despite being in remission (*P <* .01) (Figure 5*A*). The pan-specific ILC marker CD127/IL-7R was similar in UC patients and control subjects both pre- and postinjury, suggesting a specific enrichment of ILC3s in UC patients. Not only was there preinjury enrichment of the ILC3 marker in UC patients, but ILC3-activating cytokine IL-1β was induced 28.2-fold in UC patients compared with 5.3-fold in control subjects after 24 hours (*P <* .05; *P <* .01 at all time points postinjury for UC) (Figure 5*B*). Similar but less pronounced effects were found for other ILC3-activating cytokines IL-1α and IL-23, whereas the pan-ILC differentiator IL-7 was equally induced postinjury in UC patients and control subjects (Figure 5*E*). Further, UC patients had a rapidly increasing level of the ILC3-type effector cytokine IL-17A compared with control subjects (*P <* .05 and *P <* .01 for 24 and 48 hours, respectively) (Figure 5*F*). A similar but statistically insignificant rise in another ILC3-type cytokine, IL-22, was also seen (Figure 5*G*). No evidence for ILC1 or ILC2 enrichment preinjury was found.

Overall, on the one hand, the most significant differences were seen between diagnoses (control vs UC) and between preinjury and 24 hours postinjury regarding inflammatory and innate responses, whereas adaptive responses were more pronounced at 48 hours. On the other hand, control subjects had a remarkably unaffected innate and adaptive immune function in the acute injury model.

### The Microbiota of the Human Intestinal Wound Niche

**Figure 6:**
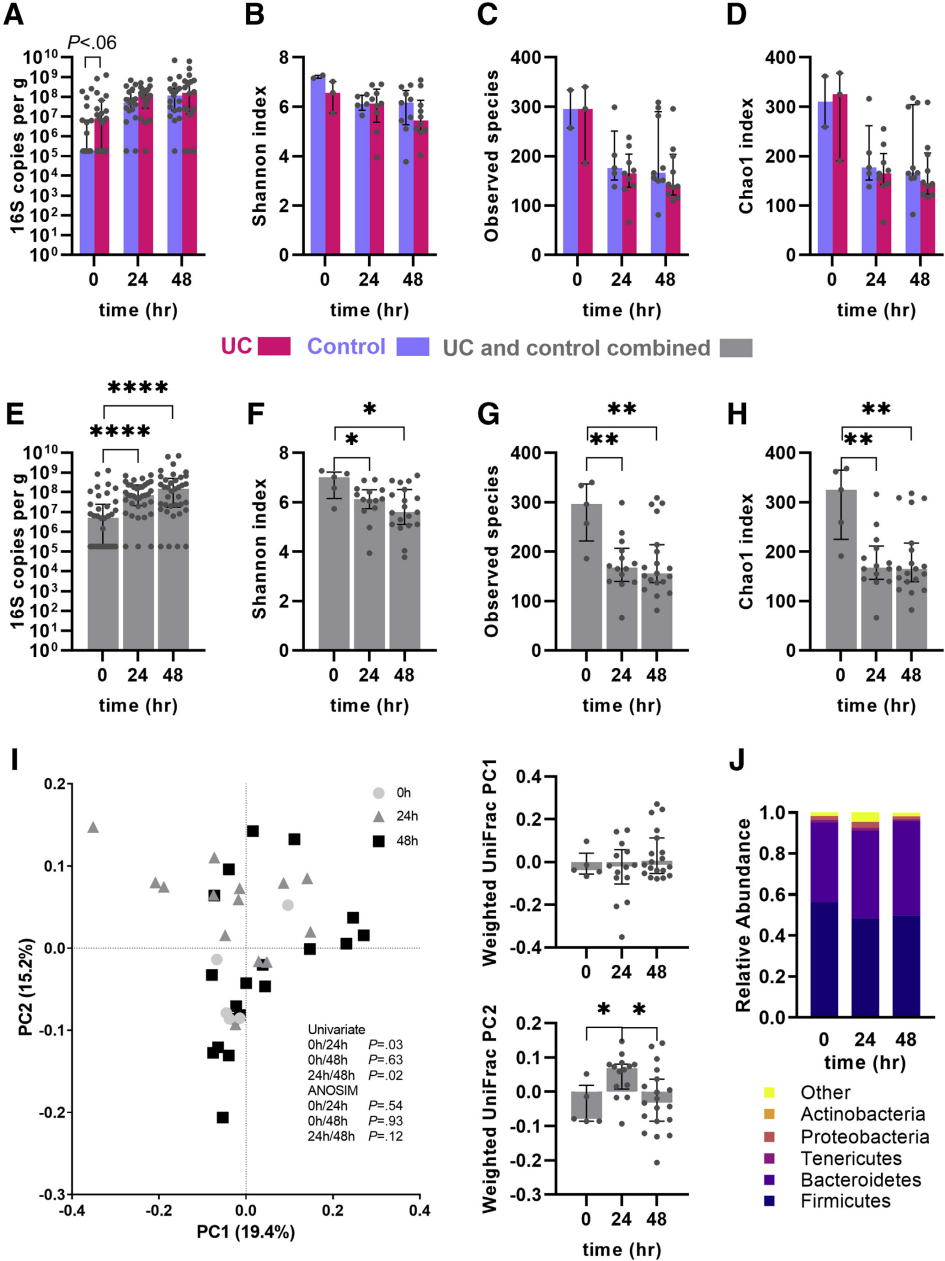
**Changes of the mucosal microbiota pre- and postinjury.** (*A–D*) Changes in (*A*) bacterial load, (*B*) α-diversity (Shannon index), (*C*) observed species number, and (*D*) estimated observed species (Chao1 index) in control and UC. (*A*) Except from a tendency towards higher mucosal bacterial load preinjury (*P =* .06), other parameters were unaffected by diagnosis (control vs UC). (*E–H*) Changes in the same parameters in the combined group of control subjects and UC patients. (*E*) An increase in bacterial load was seen along with (*F*) a decrease in α-diversity postinjury. (*G*, *H*) Similarly, the richness of species decreased significantly postinjury. (*I*) Weighted UniFrac analysis according to time for the combined control and UC cohort. While univariate analysis showed microbiome changes at 24 hours (*P <* .05), these differences were not significant using the analysis of similarities analysis (*P =* .12). Composition of phyla according to postinjury time points. Medians and interquartile ranges are shown as well as individual values. Red: UC patients. Blue: control subjects. Gray: combined control and UC. ∗*P <* .05; ∗∗*P <* .01; ∗∗∗∗*P <* .0001. PC, principal component.

Mucosa-associated bacterial load was low in the uninjured colon of both control subjects and UC patients in remission and in many instances below the limit of detection. A numerically increased bacterial load in preinjury samples in UC was seen, but this difference did not reach statistical significance (*P =* .06) (Figure 6*A*). Apart from this, injury-induced changes in bacterial load, α-diversity (Shannon index), and richness were similar in UC patients and control subjects (Figure 6*A–D*). Combined analysis of the control subjects and UC patients showed that the bacterial load in the wound niche increased 14- and 29-fold at 24 and 48 hours, respectively, after the injury compared with preinjury mucosal bacterial load (*P <* .0001) (Figure 6*E*). Concomitantly, a persistent decrease in α-diversity (Shannon index) was seen compared with the preinjury mucosal microbiome (*P <* .05) (Figure 6*F*). The decrease in diversity was accompanied by a decrease in richness in both observed and estimated (Chao1 index) number of species (*P <* .01 for all time points postinjury) (Figure 6*G* and *H*). Unweighted and weighted UniFrac analysis revealed only minor differences over time with significant instability of the microbiota composition at 24 hours postinjury as determined by univariate analysis; however, this difference was not significant using multivariate analysis of variation based on the distance matrix (Mann-Whitney *P <* .05; analysis of similarities *P =* .12) (Figure 6*I*). The data suggest that the host response changing the microenvironment of the injury niche rather than the disease per se is the major driver of dynamic changes in the microbiome of the intestine.

Only minor differences were found at phylum level postinjury (Figure 6*J*). Looking at the genus level, there was a tendency toward reduction in relative abundance in Faecalibacterium genera at 24 hours (*P =* .07, Mann-Whitney test) compared with the preinjury microbiota.

### Postinjury Host-Microbiome Interaction

**Figure 7:**
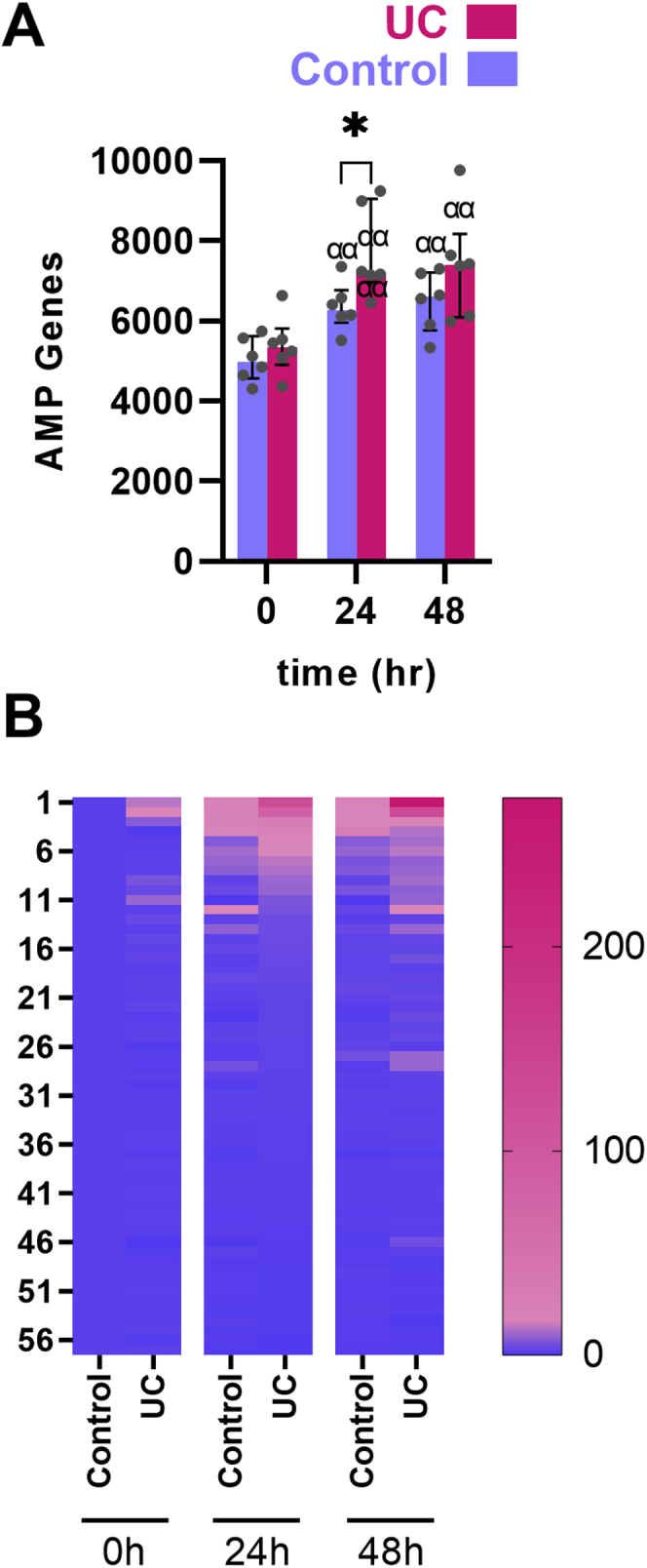
**Regulation of injury-responding AMP genes according to postinjury time points.** UC patients had higher expression of AMP genes. (*A*) The AMP gene set was developed by extracting known human proteins and peptides with antimicrobial capabilities using the Antimicrobial Peptide Database 3; see text for further description. (*B*) Heatmap of AMP gene expression. Medians and interquartile ranges are shown as well as individual values. Red: UC patients. Blue: control subjects. ∗*P <* .05. αα*P <* .01 compared with preinjury of same diagnosis.

Injury of the barrier gives access of the intestinal microbiome to the interior of the body, but this may cause interaction with immediate response elements capable of limiting the invasion. One early response could be the secretion of antimicrobial molecules and peptides. These could on the other hand modify the composition of the wound niche microbiome. To determine the postinjury antimicrobial response in humans, a gene set was developed from the Antimicrobial Peptide Database 3 consisting of 59 genes expressed in the experimental wound dataset. The injury-reacting AMPs expressed in the human intestine were induced at both time points regardless of diagnosis (*P <* .01 compared with expression levels in index biopsies from intact mucosa) (Figure 7*A* and *B*) but even more so in UC (*P <* .05 compared with control subjects at 24 hours). The most upregulated genes in UC were resisitin (*RETN*), defensin beta 4A (*DEFB4A*)*,* and serum amyloid A1 (*SAA1*), which were upregulated 3.9- to 5.3-fold in UC patients compared with control subjects (Table 3).

**Table 3:** Top Upregulated AMP Genes

Symbol	Gene name
RETN	resistin
DEFB4A	defensin beta 4A
SAA1	serum amyloid A1
CXCL3	C-X-C motif chemokine ligand 3
CXCL1	C-X-C motif chemokine ligand 1
DEFA3	defensin alpha 3
REG3A	regenerating family member 3 alpha
SAA2	serum amyloid A2
TFF2	trefoil factor 2
TFF1	trefoil factor 1
SEMG1	semenogelin 1
HAMP	hepcidin antimicrobial peptide
CCL20	C-C motif chemokine ligand 20
CXCL11	C-X-C motif chemokine ligand 11
CTSL	cathepsin L
CAMP	cathelicidin antimicrobial peptide
CTSE	cathepsin E
CCL17	C-C motif chemokine ligand 17
CXCL9	C-X-C motif chemokine ligand 9

AMP, antimicrobial peptide.

## Discussion

The experimental injury model reveals early sequential macroscopic, histopathological, and regulatory changes with concomitant wound niche microbiome changes. Importantly, early and exaggerated innate engagement is determining the difference between injury-induced hyperinflammation in UC and a balanced response in the normal colonic mucosa. Albeit the response investigated is experimental in nature, the postinjury hyperresponse could be a contributing factor in flare initiation in UC.

### Host Injury Responses

Activation of innate responses is an early event in experimental skin ulcers with recruitment of neutrophils within hours to the wound bed.^5^ Contrary to this, we found a dampened and slower innate response to injury in the normal human intestine, with no significant recruitment of neutrophils within the first 24 hours. A similarly slow and low activation pattern was found in all other aspects (innate and adaptive) of injury-related signaling in control subjects. In line with this, we found limited or no ILC2 and ILC3 signals in the normal intestine.

The ILCs are both effector cells and regulators of innate responses at mucosal surfaces. The noncytotoxic ILCs are thus termed helper ILCs and include ILC2 and ILC3 subsets, of which ILC3s are the main ILC type of the intestinal mucosa in mice.^10^ As expected, we found the pan-ILC marker IL-7R (CD127) stably and well expressed in the normal colon but with low expression of ILC2 and ILC3 activity markers.^26^

In contrast to the balanced activation found in the normal colon, patients with UC despite being in remission had a more pronounced postinjury response dominated by early neutrophil engagement. A similar quick accumulation of neutrophils postinjury was found as early as 6 hours postinjury in a study be Anthony Marks’ group.^24^ This was found to be in sharp contrast to patients with CD who has a delayed neutrophil response. Interestingly, similar findings were found using a model of acute skin injury in which heat-inactivated *Escherichia coli* was injected subcutaneously, suggesting that the changes in neutrophil recruitment to innate stimuli is not restricted to the intestine in UC.^24^^,^^27^^,^^28^ On the regulatory level, the innate hyperresponse found in our study was preceded by a preinjury UC-specific increased expression of the ILC3 marker CD117 and an early sustained increase in ILC3-stimulating cytokines IL-1β, IL-23, and IL-1α and a concomitant increased expression of IL-17A. ILC3s can be divided into 2 main subtypes dependent on the expression of the natural cytotoxicity receptor (NCR) NKp44, and the expression data suggested a balanced presence of NCR^÷^ and NCR^+^ ILC3s, consistent with a IL-17A secretory response.^9^ Supporting this notion, IL-1β and IL-23 have been found especially effective in activating ILC3s. NCR^÷^ ILC3s have recently been found to be key regulators of neutrophil recruitment through IL-17A secretion.^29^^,^^30^ Our data are consistent with the earlier finding that isolated mucosal ILC3s from patients with active UC produced more IL-22 than cells from heathy control subjects.^31^ Further, Pearson et al^32^ found that peripheral blood ILCs from patients with active IBD (not specified as UC or CD) coexpressed higher levels of IL-17A and IL-22 than healthy control subjects. The latter study also employed a murine intestinal RAR-related orphan receptor γt (RORγt)/Th17 IL-23–mediated intestinal inflammation model and reported that ILC3s were highly important for the acute phases of inflammation in this model including recruitment of monocytes, but via granulocyte macrophage colony-stimulating factor, rather than IL-17A or IL-22. Granulocyte macrophage colony-stimulating factor was found to be induced in the present acute injury model but only after 48 hours (data not shown).

Taken together, the present data are consistent with a preinjury proneness to exaggerated innate inflammation mediated through helper ILC3s. Interestingly, ILCs (and especially ILC3s) have been found to be resident in skin areas previously affected by psoriasis and chronic inflammatory lung diseases.^33^^,^^34^ It is intriguing to hypothesize that resident ILCs might explain some of the regional pattern of disease affection in both UC and CD.

Innate activation might be the initial step toward chronic inflammation as murine data suggest that dysregulated innate responses lead to adaptive immune system engagement and slower resolution of experimental colitis.^35^ Further, there are ample data suggesting a role for innate signaling in late phases of an active UC flare.^14^

### The Microbiota Mucosal Injury Niche

Accumulating data suggest that the microbiome covariates with disease and disease activity, but there is still an ongoing hen-and-egg debate on whether the changes of the microbiota causes inflammation or are secondary to the inflammation.^36^ Our data indicate that the mucosal niche microbiota are changed during acute injury of the intestine in a manner closely resembling the changes seen in UC both in terms of α-diversity, bacterial composition, and bacterial load, and that these changes are equally taking place in UC patients and control subjects. Although direct manipulation of the preinjury mucosal microbiota is not possible in the applied model, the findings suggest that microbiota changes are secondary to the inflammation induced by the mucosal injury and a normal pathophysiological reaction of the microbiota-host interaction during mucosal injury, as opposed to the disease-specific innate response changes in UC discussed previously. Interestingly, as in active UC, we find a tendency for a decrease in *Faecalibacterium spp.* abundance in the wound niche at 24 hours compared with preinjury.^37^ Further, there was a nonsignificant tendency toward increased mucosal bacterial load in our cohort in the preinjury samples from UC patients in line with early studies on the role of host-microbiota interaction in UC.^38^

## Conclusion

By looking at responses to an experimental injury of the human intestine, we found that the normal injury response is a dampened with limited innate and adaptive engagement and rapid induction of regenerative pathways. In contrast, patients with quiescent UC have an unconstrained hyperresponsive innate response pattern associated with increased macroscopic and histopathologic inflammation. Our data may add evidence to the dynamics of inflammation-dependent de-diversification of the IBD microbiota and may not only explain some of the mechanisms behind flare initiation in UC, but also provide a functional model and molecular platform for the development of injury response–modifying therapies.

## Materials and Methods

### Study Population

Nineteen patients with quiescent UC and 20 control subjects were included in the study (Table 1). Patients were in clinical remission defined as a total Mayo score <3 and an MES of 0. Patients 18–70 years of age could participate, and the UC diagnosis was established according to international criteria at least 12 months prior to inclusion.^39^ Oral mesalazine or azathioprine on stable dosing for 6 months was allowed, whereas antibiotics, systemic glucocorticoids, biologicals, nonsteroidal anti-inflammatory drugs, and other immune-related medication within the past 6 months were excluded. Lactating or pregnant women and patients with malignancies were excluded.

### Injury Assay

The injury assay was a development of earlier wound assays^24^^,^^25^ and the method is outlined in Figure 1*A*. Patients had an initial sigmoidoscopy where the MES was determined. During the procedure, 6 experimental wounds (size 7.0 × 1.5 mm) were made using a 2.8-mm biopsy forceps (Radial Jaw 4; Boston Scientific, Marlborough, MA) with a distance of at least 2 cm from each other: Two samples were fixed in paraformaldehyde, 2 were snap-frozen in liquid nitrogen and subsequently stored for 16s ribosomal RNA (rRNA) gene analysis, and 2 were stored in RNA later (Thermo Fisher Scientific, Waltham, MA) at 4°C for 24 hours and then –80°C. After 24 and 48 hours, the subjects were re-endoscoped and the initial wounds were identified and documented by high-definition video recording (Olympus Evis Exera III platform; Olympus, Tokyo, Japan). Wound biopsies were taken across the experimental injury by angling the biopsy forceps 90° (see Figure 1), thus including the mucosa of the injury edge and the wound bed using 3 different wounds each time. The wound biopsies were stored in the same way as described previously.

### Wound Score

Based on the knowledge from skin excision wound models,^40^ a scoring system was developed taking into account barrier breach exudate appearance and signs of inflammation (peripheral edema and erythema) and wound healing (closure of defect) (see Table 2). Wounds were scored blindly and independently by 2 gastroenterologists (J.B.S. and J.T.B.), and the mean wound score was used.

### Assessment of Histological Inflammation

Inflammation was assessed using the validated Geboes score.^41^ The score grades inflammation and structural changes associated with UC on a scale from 0.0 to 5.4 and is used as a standard score to assess mucosal healing in UC clinical trials. Because ulceration of the mucosa automatically assigns the highest grade in the score and all the samples had ulcers from the experimental wound, a modified Geboes score was used, leaving out erosion or ulceration. The modified Geboes score thus ranges from 0.0 to 4.4. All biopsies were scored blindly by an IBD pathologist (L.B.R.).

### 16S rRNA Gene Sequencing

All subjects had 16S rRNA sequencing performed at all time points. The QIAamp DNA Stool Mini Kit (Qiagen, Hilden, Germany) was used according to the manufactures protocol with the modification that each sample was added 0.70 mm Garnet Beads (MO BIO Laboratories, Carlsbad, CA) and vortexed for 10 seconds, and a second bead-beating was performed following the heating at 70°C for 5 minutes of the suspension in 0.1-mm Glass Bead Tubes (MO BIO Laboratories), and vortexed for 10 minutes to increase the yield of bacterial DNA. Bacterial load was determined by quantitative polymerase chain reaction (qPCR) of 16S rDNA relative to a purified *E. coli* K12 DNA standard.

16S rRNA sequencing was performed via the MIT BioMicroCenter. To prepare sequencing libraries, samples were amplified over 25 PCR cycles (Phusion reagent kit; New England Biolabs, Ipswich, MA) (primers—forward: ACACGACGCTCTTCCGATCTYRYRGTGCCAGCMGCCGCGGTAA; reverse: CGGTCTCGGCATTCCTGCTGAACCGCTCTTCCGATCTGGACTACHVGGGTWTCTAAT; Integrated Data Technologies, Newark, NJ) and purified with Agencourt AMPure XP SPRI beads (Beckman Coulter, Brea, CA). Samples were normalized by qPCR (SYBR green; MilliporeSigma, Burlington, MA) and amplified for an additional 7 PCR cycles, SPRI purified, qPCR quantified, and normalized to lowest concentration. Samples were then pooled on an equimolar basis, and run on a MiSeq (Illumina, San Diego, CA) 250 PE run. MiSeq image analysis and base calling was carried out by RTA 2.5.1.3 with a single FASTQ as end product. BioMicroCenter pipeline release 1.5.2 was run to demultiplex FASTQs and generate quality control metrics.

### RNA Sequencing

A randomly selected subset of 6 UC patients and 6 control subjects had RNA sequencing performed on all time points. Biopsies kept in RNA later were transferred to a lysis buffer containing 2% mercaptoethanol (Sigma-Aldrich, St Louis, MO), homogenized, and total RNA was extracted using Nucleospin columns (Macherey-Nagel, Düren, Germany). The quantity and purity of the RNA was assessed on a Nanodrop ND-1000 spectrophotometer (Thermo Fisher Scientific) and quality by 2100 Bioanalyzer (Agilent Technologies, Santa Clara, CA; RIN range 8.1–9.5). RNA sequencing (Illumina PE150 system; Illumina) was performed by Novogene (Cambridge, United Kingdom), and bioinformatic analyzes were subsequently conducted at BRIC (University of Copenhagen) and the Koch Institute for Integrative Cancer Research (MIT). The raw reads were quality assessed with FastQC and FastQ Screen^42^ and trimmed using Trimmomatic (v.0.32).^43^ The trimmed reads were aligned to the hg38 genome assembly using STAR (v.2.5.1a)^44^ in 2-pass mode and guided by a RefSeq (UCSC, 2018.08.05) gene annotation.

After mapping, reads were assigned to genes using featureCounts (v.1.5.1),^45^ thereby generating a count table. In R (v.3.5.1; R Foundation for Statistical Computing, Vienna, Austria), the DESeq2 (v.1.22.1)^46^ package was used for statistical analysis of the count data.

### RNA-sequencing data are available at the Gene Expression Omnibus with accession number GSE164918

#### Analysis Strategy

A principal component analysis plot was generated using the top 500 genes with the most variable counts across samples. The gene set enrichment analysis function in clusterProfiler R package^47^ was used to test for under- or overrepresentation of genes in various gene sets from the MSigDB database based on the normalized log fold changes.

To infer changes in signaling pathway activity of the wound site associated with time and diagnosis, we applied the PROGENy signaling pathway inference package^48^ to variance-stabilized count data fitted by a group model through DESeq2 (each level of the group corresponding to a unique time × diagnosis). Pairwise *t* testing with Benjamini-Hochberg multiple comparison correction was performed over all groups, with significance at adjusted *P <* .05.

Downstream bioinformatics analysis of the microbiome was performed in the QIIME2 pipeline.^49^ A total of 38 samples (0 hours: 5; 24 hours: 14; and 48 hours: 19) with a median of 58,483 reads (range, 16,156–197,521) were included in the analysis. Alpha diversity was assessed by the Shannon Diversity Index and enrichment determined by observed species analysis and the Chao1 index.^50^ The bacterial community composition was analyzed at the phylum level.

Mean gene expressions within gene sets of relevance for acute injury reactions (GO:0045087 Innate immune response, GO:002821 Adaptive immune response, and GO:0042246 Tissue regeneration) were assessed by nonparametric Mann-Whitney test (significance at *P <* .05). Further, a gene set consisting of 59 genes (Table 3) was developed by extracting known human proteins and peptides with antimicrobial capabilities using the Antimicrobial Peptide Database 3.^51^ A gene set consisting of 6 chemokines recruiting neutrophils (CCL2, CCL3, CXCL1, CXCL2, CXCL5, CXCL8) and T and B cells (CCL20, CCL24, CXCL1, CXCL12) was constructed.

Data were expressed as medians with interquartile ranges. To compare 2 groups, the Wilcoxon test for paired or Mann-Whitney for unpaired data were applied using GraphPad 9.0.0 (GraphPad Software, San Diego, CA). A significance level of *P <* .05 was applied.
